# Fixed Differences in the *paralytic* Gene Define Two Lineages within the *Lutzomyia longipalpis* Complex Producing Different Types of Courtship Songs

**DOI:** 10.1371/journal.pone.0044323

**Published:** 2012-09-07

**Authors:** Rachel M. M. A. Lins, Nataly A. Souza, Reginaldo P. Brazil, Rhayza D. C. Maingon, Alexandre A. Peixoto

**Affiliations:** 1 Laboratório de Biologia Molecular de Insetos, Instituto Oswaldo Cruz, FIOCRUZ, Rio de Janeiro, Brazil; 2 Laboratório de Transmissores de Leishmanioses, Instituto Oswaldo Cruz, FIOCRUZ, Rio de Janeiro, Brazil; 3 Laboratório de Bioquímica e Fisiologia de Insetos, Instituto Oswaldo Cruz, FIOCRUZ, Rio de Janeiro, Brazil; 4 Centre for Applied Entomology and Parasitology, Institute for Science and Technology in Medicine, School of Life Sciences, Keele University, Keele, United Kingdom; 5 Instituto Nacional de Ciência e Tecnologia em Entomologia Molecular (INCT-EM), Rio de Janeiro, Brazil; University of Arkansas, United States of America

## Abstract

The sand fly *Lutzomyia longipalpis* (Diptera: Psychodidae: Phlebotominae), the most important vector of American visceral leishmaniasis, is widely distributed in Latin America. There is currently a consensus that it represents a species complex, however, the number and distribution of the different siblings is still uncertain. Previous analyses have indicated that Brazilian populations of this vector can be divided into two main groups according to the type of courtship song (Burst vs. Pulse) males produce during copulation. Nevertheless, no diagnostic differences have been observed between these two groups with most molecular markers used to date. We analyzed the molecular divergence in a fragment of the *paralytic* (*para*) gene, a locus involved in the control of courtship songs in *Drosophila*, among a number of *Lu. longipalpis* populations from Brazil producing Burst and Pulse-type songs. Our results revealed a very high level of divergence and fixed differences between populations producing the two types of songs. We also compared *Lu. longipalpis* with a very closely related species, *Lutzomyia cruzi*, which produces Burst-type songs. The results indicated a higher number of fixed differences between *Lu. cruzi* and the Pulse-type populations of *Lu. longipalpis* than with those producing Burst-type songs. The data confirmed our previous assumptions that the presence of different sibling species of the *Lu. longipalpis* complex in Brazil can be divided into two main groups, one representing a single species and a second more heterogeneous group that probably represents a number of incipient species. We hypothesize that *para* might be one of the genes directly involved in the control of the courtship song differences between these two groups or that it is linked to other loci associated with reproductive isolation of the Brazilian species.

## Introduction

The study of species complexes provides an opportunity to investigate a number of unanswered questions about speciation [1]. Divergence between populations resulting in recent or incipient speciation can eventually lead to a number of molecular, behavioral and morphological changes, but very often these characters do not evolve at similar rates. This is particularly true in cases of cryptic speciation [2] where morphologically indistinguishable species can show striking behavioral differences, especially in aspects of courtship.

Acoustic communication is an important aspect of sexual behavior in a large number of insects [3], including disease vectors (e.g. [4]–[6]), and it has also a role in the reproductive isolation of many closely related species. In *Drosophila,* for example, courtship song is usually species-specific, being one of the signals females use to recognize males of their own species (e.g. [7]–[10]). *Drosophila* studies have also identified a number of genes controlling features of courtship songs (reviewed by [11]–[12]).

Acoustic signals can be also useful as one of the markers in an integrative analysis for species identification where classic morphologic differences fail to differentiate incipient sibling species [13]. One example in blood-sucking insects involves study of male copulation songs in the *Lutzomyia longipalpis* species complex [14]–[16], the main neotropical vector of *Leishmania infantum*, the etiological agent of American visceral leishmaniasis (AVL) [17]. As the main vector of an important parasitic disease, the existence of cryptic species in this insect may have important epidemiologic consequences [18]–[19] since divergence caused by genetic drift and/or natural selection may affect genes controlling aspects of the disease vector potential, resulting in sibling species that are more efficient as vectors than others as has been shown in the *Anopheles gambiae* species complex [20]–[21].

Although *Lu. longipalpis* is a species complex [22]–[25], the number and distribution of the different sibling species is still uncertain. Previous studies using a combination of crossing experiments [22], [26], analyses of acoustic signals [14]–[16], male sex pheromones [16], [27]–[30] and molecular markers including orthologues of *Drosophila* courtship song genes *period* (*per*) and *cacophony* (*cac*) [16], [31]–[33], and microsatellites [34]–[35], have indicated that the Brazilian populations of this vector can be divided into two main groups according to the type of copulation song (Burst vs. Pulse) and pheromones that males produce [16]. Males of the first group of populations produce Burst-type song and the diterpene Cembrene-1 pheromone and probably represent a single species while the second group consists of populations producing different subtypes of Pulse-type song in combination with different pheromones that probably represent a number of incipient species [16]. However, *Lu. longipalpis* genetic structure in Brazil is rather complex with evidence of incomplete reproductive isolation and introgression [16], [22], [33] and no observed diagnostic differences between these two groups in most molecular markers used so far that would allow for a rapid identification of the different species.

The only potential exception so far is the *paralytic* (*para*) gene, a locus also involved in the control of courtship songs in *Drosophila* [36], characterized by fixed differences between a pair of sympatric sibling species of the *Lu. longipalpis* complex from Sobral (Ceára State, Brazil), that produce different copulation songs and male sex pheromones [37]. In the present study, we have extended the analysis of the *para* gene to a number of other *Lu. longipalpis* populations from Brazil. In addition, we have also analyzed the differentiation between *Lu. longipalpis* and *Lutzomyia cruzi*, a closely related species [38] that also acts as a vector of *Le. infantum* in a region of Brazil [39]. Analyses of copulation songs, pheromones and molecular markers have indicated that *Lu. cruzi* is another species of the *Lu. longipalpis* complex [35], [40]–[41].

## Methods

**Figure 1:**
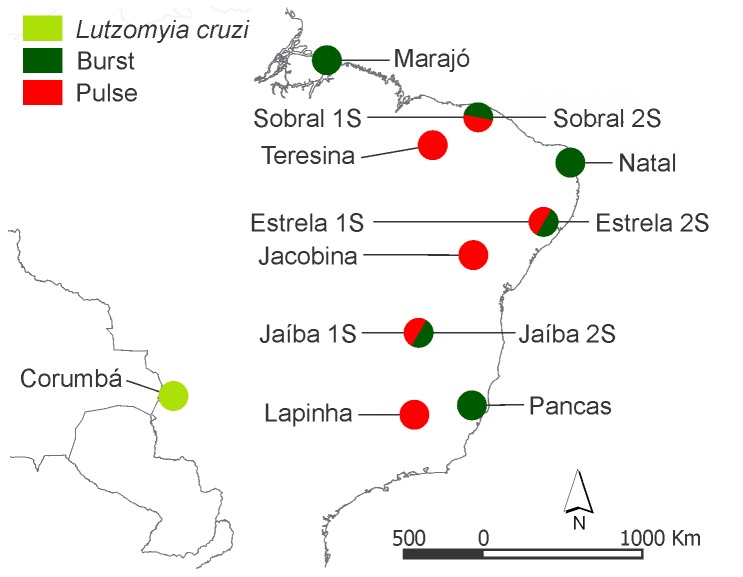
Map of Brazil with the approximate location of the studied samples.

We analyzed samples of *Lu. longipalpis* from eight different Brazilian localities: Lapinha, Minas Gerais State; Jaíba, Minas Gerais State; Jacobina, Bahia State; Pancas, Espírito Santo State; Estrela de Alagoas, Alagoas State; Natal, Rio Grande do Norte State; Marajó Island (Salvaterra), Pará State; and Teresina, Piauí State (Figure 1). A permit for sand fly collection in Brazil was obtained from the Brazilian Ministry of Environment (SISBIO #26066-1). Sand flies were captured using CDC light-traps near human habitation with permission from local homeowners. In addition, the collections were usually supported by the local vector surveillance authorities from local State Health Departments. Male *Lu. longipalpis* are characterized by polymorphism in the number of abdominal spots [22]: although this phenotype cannot be used to identify different allopatric species of the complex, it can be useful in some cases of sympatry, as previous work in Sobral (reviewed in [19], [25]) and, more recently, in Estrela de Alagoas and Jaíba [16] has shown. In these three localities, the sympatric one spot (1S) and two spot (2S) males produce different copulation songs (Pulse-type and Burst-type, respectively) and represent different species [16]. Therefore, these samples were analyzed separately. Males from Natal that are highly polymorphic for the number of spots including very high numbers of intermediate forms which are rare in Sobral, Estrela de Alagoas and Jaíba, and Pancas (1S) produced Burst-type song, while males of Lapinha (1S), Jacobina (2S) and Teresina (1S) that represents a majority of this locality produce different subtypes of Pulse-type song [16]. We also analyzed a sample of *Lu. cruzi* from Corumbá, Mato Grosso do Sul State and two males of *Lutzomyia pseudolongipalpis* from Curarigua, Venezuela, used as an outgroup in the genealogical analysis.

Genomic DNA was isolated according to [42] and the PCR Master Mix (Promega) was used to perform PCR according to [37]. PCR products were purified using the Wizard SV Gel and PCR Clean-up System (Promega) or GFX PCR DNA and Gel Band Purification Kit (GE Healthcare). Purified fragments were cloned using the pMOS*Blue* Blunt Ended Cloning Kit (GE Healthcare) or TOPO TA Cloning Kit (Invitrogen). Plasmid DNA was isolated using Flexiprep Kit (GE Healthcare) or using 96 well microplates and the alkaline lysis method [43] followed by filtration in Millipore Multiscreen filter plates. DNA sequencing was carried out with an ABI 3730 sequencer using the Big Bye 3.1 Kit (Applied Biosystems).

*Lu. longipalpis para* gene fragments from all populations were initially processed using BioEdit Sequence Alignment Editor [44] before population genetics analyses, which also included previously published sequences from Sobral [37]. A minimum of eight sequences per individual were aligned to obtain two consensus sequences corresponding to the two alleles, A and B, or one consensus sequence where flies were treated as homozygotes and the sequences were duplicated. The estimated probability of misclassifying a heterozygous fly as a homozygous with this procedure was less than 1%.

Both polymorphism and population structure analyses were carried out using DnaSP v5 [45] and Proseq 2.91 [46]. A Minimum Evolution tree based on p distances was estimated using MEGA5 [47]. All sequences were submitted to GenBank (accession numbers JQ359112–JQ359437). Analysis of molecular variance (AMOVA) was carried out with Arlequin 3.11 [48]. A non-recombinant block of the initial fragment was obtained using the IMgc program [49] to construct the haplotype network with TCS v1.21 software [50].

## Results

We analyzed a total 298 allele sequences from 149 males of a fragment of the *para* gene of *Lu. longipalpis* [37] of approximately 385 bp, including a variable sized intron of ∼220 bp. Analyses included previously published and new sequences from the two Sobral sympatric sibling species [37] and new sequences from samples of the eight Brazilian localities analyzed here (Figure 1). Sympatric one spot (1S) and two spot (2S) males found in Estrela de Alagoas and Jaíba were analyzed separately since these males produce different copulation songs (Pulse and Burst, respectively) and represent different species, as previously observed in Sobral [16]. We also analyzed 24 allele sequences of *Lu. cruzi* males from Corumbá, State of Mato Grosso do Sul, a closely related sibling of the *Lu. longipalpis* complex producing Burst-type song [41], and used 4 sequences obtained from two males of *Lu. pseudolongipalpis*, a more distantly related sibling species [35], [51], as an outgroup in the genealogical analysis (see below). Figure S1 shows the alignment of the whole fragment. Most of the variation was found within the intron, that included a number of indels. However, some rare non-synonymous substitutions were also observed.

Table 1 shows a summary of the polymorphisms for each population analyzed, excluding the regions with gaps. Populations of *Lu. longipalpis* were grouped according to the type of copulation song they produce: Pulse or Burst. The results showed that Lapinha was the least polymorphic among the Pulse song populations while Jacobina had the highest values of ð and è. Among the Burst song populations, Marajó and Jaiba 2S were the least and most polymorphic samples, respectively. Tajimás D and Fu and Li's D* and F* tests of neutrality [52]–[53] were performed for each population. Although one value was significant at a 5% level, all values were non-significant after Bonferroni correction.

**Table 1:** Polymorphism summaries of the *para* gene fragment from populations of *Lu. longipalpis* and *Lu. cruzi*.

Population	Song-type	n	S	ð	è	D_T_	D*	F*
Sobral 1S	P	32	12 (12)	0.0033 (0.0032)	0.0080 (0.0079)	−2.0334*	−1.2001	−1.7165
Lapinha	P	28	2 (2)	0.0016 (0.0016)	0.0014 (0.0014)	0.3094	−0.7144	−0.4930
Jacobina	P	22	9 (9)	0.0051 (0.0050)	0.0067 (0.0066)	−0.8129	−1.2837	1.3311
Teresina	P	24	9 (9)	0.0037 (0.0036)	0.0065 (0.0064)	−1.4200	−2.4491	−2.4955
Jaíba 1S	P	24	6 (6)	0.0023 (0.0023)	0.0043 (0.0043)	−1.3944	−0.9729	−1.2699
Estrela 1S	P	22	5 (5)	0.0019 (0.0019)	0.0037 (0.0037)	−1.4525	−0.4601	−0.8577
Sobral 2S	B	28	8 (9)	0.0040 (0.0057)	0.0056 (0.0073)	−0.8846	0.0821	−0.2423
Estrela 2S	B	32	6 (7)	0.0038 (0.0056)	0.0041 (0.0058)	−0.1481	1.2092	0.9335
Jaíba 2S	B	24	11 (11)	0.0047 (0.0047)	0.0080 (0.0080)	−1.3831	−1.3688	−1.5994
Natal	B	24	11 (12)	0.0047 (0.0049)	0.0080 (0.0087)	−1.4081	−1.8366	−1.9906
Pancas	B	32	10 (11)	0.0047 (0.0051)	0.0067 (0.0074)	−0.9295	−1.3358	−1.4167
Marajó	B	6	1 (3)	0.0016 (0.0048)	0.0012 (0.0044)	−1.7188	1.0525	1.1577
*Lu. cruzi*	B	24	5 (5)	0.0019 (0.0018)	0.0037 (0.0036)	−1.4315	−2.1728	−2.2703

**B.** Burst type song; **P.** Pulse type song; **n**. number of sequences; **S**. number of segregating sites; **ð**. nucleotide diversity; **è**. neutral parameter based on the segregating sites; **D_T_**. Tajima test of neutrality. D* and F*. Fu and Li's tests of neutrality. Numbers in parentheses represent the analysis of nucleotide diversity considering the regions with gaps. *p<0.05.

Molecular differentiation analysis was performed for all pairwise comparisons involving the *Lu. longipalpis* populations, except for the small sample of Marajó. Again, the populations were grouped according to the song type they produce, Pulse or Burst. Table 2 shows the fixation indexes (Fst) as well as the number of fixed differences (Sf) in each comparison. The lowest pairwise Fst values were obtained between populations producing the same song type, while very high values of differentiation were observed in the comparisons involving populations producing either Burst or Pulse-type copulation songs. Indeed, fixed differences were found in those latter comparisons, except for the Estrela 1S sample. However, when sequences of a single fly (sequences Est1S8A and Est1S8B) were excluded from the analysis, this Pulse song population also showed fixed differences when compared to all other Burst song populations (numbers within brackets). Previous analysis by Araki et al. [16] suggested that the spot phenotype in this locality might not be as reliable for identifying the two sympatric sibling species as in Sobral [15], [22], [30], [32]–[34], [37]. However, *para* gene Fst values clearly confirm the presence of two sympatric species in Estrela, i.e. Estrela 1S and 2S (Table 2 and below).

**Table 2:** Pairwise differentiation between Pulse-type and Burst-type populations of *Lu. longipalpis* and *Lu. cruzi*.

		[Pulse-type populations]						[Burst-type populations]					
		S1S	Lap	Jac	Ter	J1S	E1S	S2S	E2S	J2S	Natal	Pancas	*Lu. cruzi*
**Pulse-type**	**S1S**		0.2077^***^	0.1261^**^	0.0315^ns^	0^ns^	0.6171^***^	0.7819^****^	0.8257^****^	0.8103^****^	0.8009^****^	0.8003^****^	0.8695^****^
**populations**							(0.4914^****^)						
	**Lap**	0		0.2915^***^	0.2633^*^	0.2007^*^	0.8079^***^	0.8285^****^	0.8704^****^	0.8504^****^	0.8455^****^	0.8443^****^	0.9083^****^
							(0.6491^***^)						
	**Jac**	**0**	**0**		0.1697^**^	0.1706^**^	0.3251^**^	0.6899^****^	0.7414^***^	0.7335^***^	0.7146^****^	0.7152^****^	0.8002^***^
							(0.2343^**^)						
	**Ter**	0	0	0		0.0050^ns^	0.6002^***^	0.7701^****^	0.8122^***^	0.7987^***^	0.7886^***^	0.7883^****^	0.8558^****^
							(0.4938^***^)						
	**J1S**	0	0	0	0		0.7031^***^	0.8026^****^	0.8458^***^	0.8282^****^	0.8209^****^	0.8200^****^	0.8871^***^
							(0.5603^***^)						
	**E1S**	0 (0)	1 (1)	0 (0)	0 (0)	1 (1)		0.8356^***^	0.8913^**^	0.8627^**^	0.8579^**^	0.8561^***^	0.9271^**^
								(0.7312^***^)	(0.7922^**^)	(0.7741^***^)	(0.7581^***^)	(0.7577^****^)	(0.8504^**^)
**Burst-type**	**S2S**	3	4	2	3	4	3 (0)		0.0635^ns^	0.2272^**^	0.0380^ns^	0.0914^*^	0.7139^****^
**populations**													
	**E2S**	3	4	2	3	4	3 (0)	0		0.0744^ns^	0^ns^	0^ns^	0.7761^***^
	**J2S**	3	4	2	3	4	3 (0)	0	0		0.0737^ns^	0.0633^*^	0.7534^****^
	**Natal**	3	4	2	3	4	3 (0)	0	0	0		0^ns^	0.7387^****^
	**Pancas**	3	4	2	3	4	3 (0)	0	0	0	0		0.7364^****^
	***Lu. cruzi***	5	6	4	5	6	5 (2)	2	2	2	2	2	

Upper right matrix – pairwise differentiation (Fst) and significance (P values were obtained with 10,000 random permutations). Lower left matrix – fixed differences between samples. S1S – Sobral 1S, Lap – Lapinha, Jac – Jacobina, Ter – Teresina, J1S – Jaíba 1S, S2S – Sobral 2S, E2S – Estrela 2S, J2S – Jaíba 2S. S1S – Sobral 1S. Lap – Lapinha. Jac – Jacobina. Ter – Teresina. J1S – Jaíba 1S. S2S – Sobral 2S. E2S – Estrela 2S. J2S – Jaíba 2S. Significance of pairwise Fst values was estimated with 10,000 random permutations. Values between brackets included the single E1S fly which probably represents a case were the spot phenotype does not match the song type in this population.

ns - non-significant; *p<0.05; **p<0.01; ***p<0.001; ****p<0.0001.

Smaller sequence differences in *para* were observed between Burst-type populations than between Pulse-type populations. Indeed, the mean pairwise Fst value among Burst-type populations was 0.063
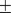
0.067 compared with 0.147
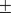
0.104 among Pulse-type populations. In contrast, the mean pairwise differentiation between populations with the two main song types was much higher (0.790
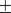
0.044). These results were corroborated by AMOVA performed to examine the partition of *para* sequence variation within *Lu. longipalpis* (Table 3). Most of the molecular variation (64.95%) was observed between the two main song types (Burst × Pulse), reflecting a clear separation between these groups. In addition, the results revealed a small part of this variation (7.0%) distributed among populations within groups.

**Table 3:** AMOVA statistics.

Source of variation	Percentage of variation
Among groups	64.95
Among populations within groups	7.00
Within populations	28.06
Fsc (haplotypes/populations within groups)	0.1996^***^
Fst (haplotypes/populations/groups)	0.7194^***^
Fct (populations/groups)	0.6495^*^

**Copulation song groups:** **Burst-type:** Sobral 2S, Estrela 2S, Jaíba 2S, Natal and Pancas; **Pulse- type:** Sobral 1S, Jaíba 1S, Estrela 1S, Lapinha, Jacobina and Teresina. Significance corresponding to the fixation indexes was obtained through 10,000 permutations. *p<0.01; ***p<0.0001.

The same 383 bp *para* gene fragment studied in *Lu. longipalpis* was also amplified in *Lu. cruzi* from Corumbá, State of Mato Grosso do Sul. As shown in Table 1, *Lu. cruzi* showed levels of polymorphism in *para* that were similar to the lowest values observed among the *Lu. longipalpis* samples. Higher differentiation and fixed nucleotide differences between *Lu. cruzi* and all *Lu. longipalpis* populations with high Fst values (ranging from 0.7139 and 0.9271) were also observed (Table 2). However, a number of Fst values were smaller than comparisons between Burst-type and Pulse-type populations of *Lu. longipalpis*. Furthermore, *Lu. cruzi* that produces Burst-type songs showed two fixed differences compared with Burst-type populations and four to six differences compared with Pulse-type populations, whereas comparisons between the two song types of *Lu. longipalpis* displayed two to four fixed differences.

**Figure 2:**
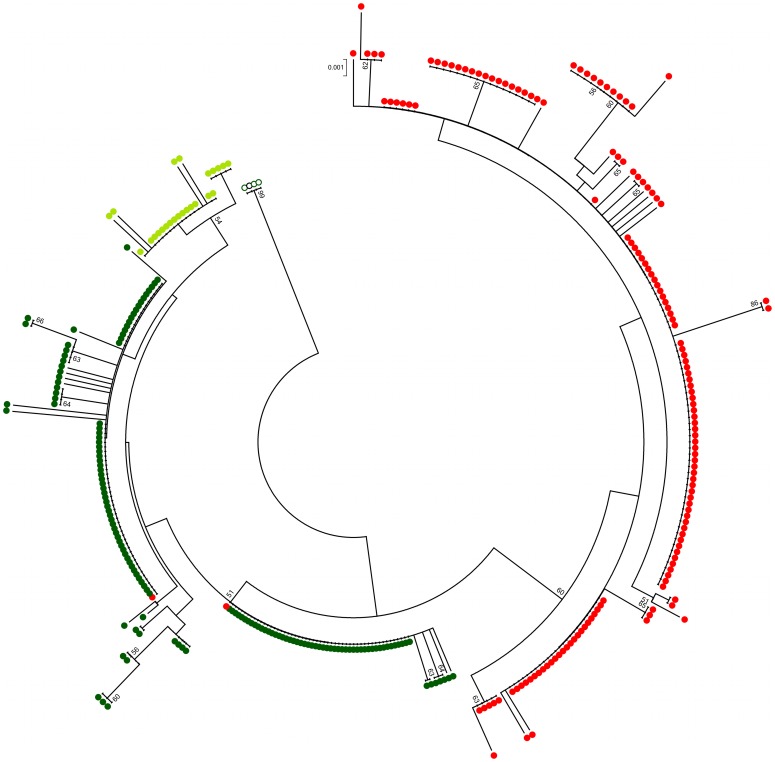
Minimum Evolution tree of sequences from Brazilian populations of *Lu. longipalpis* producing Burst-type (dark green circles) and Pulse-type songs (red circles), *Lu. cruzi* (light green circles) and the more distant sibling species *Lu. pseudolongipalpis* (open circles) used as outgroup. The sequences E1S8A and E1S8B are the only red circles that cluster with the Burst-type sequences. Bootstrap values based on 1000 replications (values below 50% are not shown).

A Minimum Evolution tree including all *Lu. longipalpis* and *Lu. cruzi* sequences and those from the more distant sibling *Lu. pseudolongipalpis* (Figure 2) showed clear separation between the two main groups producing different copulation songs. In the tree, the two sequences (E1S8A and E1S8B) belonging to one Estrela de Alagoas 1S fly that were excluded from the Fst analysis (Table 2) clustered with the sequences corresponding to the Burst-type populations indicating that this individual probably represents a case where the spot phenotype did not match the correct song type in this locality. Sequences of *Lu. cruzi* that also produce Burst-type songs (light green circles) were grouped together with the *Lu. longipalpis* Burst-type sequences. As expected, *Lu. pseudolongipalpis* (open circles) were isolated from all other populations.

**Figure 3:**
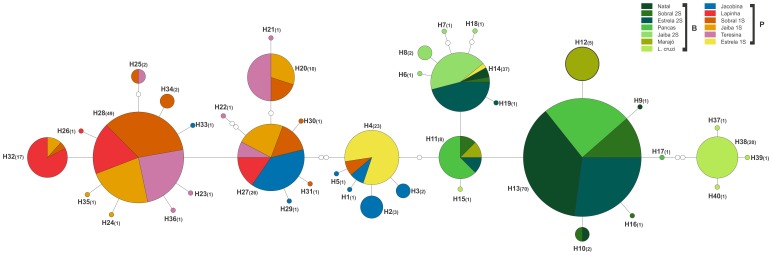
Haplotype network of Brazilian populations of *Lu. longipalpis* and *Lu. cruzi*. Each population is represented by a different color and each node represents a unique haplotype.

Finally, a haplotype network (Figure 3) was constructed based on a 249 bp non-recombinant fragment generated from the original segment of the *para* gene to avoid ambiguities due to recombinant events. A total of 40 haplotypes with 37 segregating sites were identified (Table S1) and a single network was generated using a 95% connection limit, except for *Lu. pseudolongipalpis*, which did not group in the same network.

The two main haplotypes generated were H13 and H28. Haplotype 13 corresponds to Burst-type populations and was composed of sequences of Sobral 2S, Estrela 2S, Natal and Pancas. Haplotype 28 was the most frequent haplotype of Pulse song populations from Sobral 1S, Jaíba 1S, Lapinha and Teresina. There was clear separation between the two groups producing different song types. These groups were connected by a single mutation between H11 and H4. H11 represents sequences of Sobral 2S, Pancas, Estrela 2S and Marajó. Interestingly, most of the sequences corresponding to H4 are from Estrela 1S, whose males produced the same type of pheromone, Cembrene-1 [16] found in populations with the H11 haplotype. In addition, nearly all *Lu. cruzi* haplotypes appeared as a separate cluster more closely related to the Burst-type populations of *Lu. longipalpis.*

## Discussion

Understanding the structure of sibling species complexes is a difficult task for evolutionary biologists and this is particularly true in the case of cryptic species [2]. The lack of diagnostic morphological changes coupled with incomplete reproductive isolation and introgression, a common phenomenon among very closely related siblings [54]–[55], makes the identification and delimitation of the different species a difficult assignment.

Combined analyses using molecular markers, particularly the *per* gene [16] and microsatellites [34]–[35], and behavioral traits (songs and pheromones) strongly suggest that Brazilian *Lu. longipalpis* populations can be divided into two main groups according to the type of song (Burst vs. Pulse) males produce during copulation [16]. Fixed *para* gene differences between these two main lineages further support this notion. Indeed, the haplotype networks obtained with *per* [16] and *para* (Fig 3) showed a clear separation between the two population groups. In addition, although no fixed differences between the two lineages were observed in *per*, the pairwise divergence between *Lu. longipalpis* populations measured by Fst values in these two genes were highly correlated (Mantel test, r = 0.819, p<0.01). Furthermore, both genes show a higher level of divergence among Pulse-type than among Burst-type song populations, consistent with the idea that the latter populations that produce the same song-type and the same pheromone (Cembrene-1) belong to a single species [16]. However, data from both genes indicate that the relationship among populations producing the different subtypes of Pulse-type song is more complex and heterogeneous. For example, males from Jacobina produce the P1 song and a combination of alpha-himachalene and 3-methyl-alpha-himachelene sex pheromones; Lapinha males produce the P2 song and 9-methyl-germacrene-B, (9MGB), sex pheromone; and Sobral 1S and Teresina produce the same P3 song associated with 9MGB sex pheromone [16]. Jaíba 1S males produce the P4 song and Cembrene-2 sex pheromone whereas in Estrela, 1S males produce the P5 song and the Cembrene-1 sex pheromone. Thus, combined molecular and behavioral data strongly suggest that these populations belong to five different incipient sibling species [16]. Indeed, for at least one pair of Pulse-type song populations (Jacobina and Lapinha) crossing experiments [26] and cytogenetic analysis [56] support this hypothesis.

Comparative *para* and *per* data ( [41], this study) also suggest that *Lu. cruzi* is another member of the *Lu. longipalpis* complex. However, *per* analysis indicated higher genetic differentiation between *Lu. cruzi* and Burst-type song populations where the present results with *para* showed a higher Fst value between the former and Pulse-type populations. *Lu. cruzi* males produce a variation of the Burst-type song with shorter inter-burst intervals [41] and the 9MGB sex pheromone [40] also found in many Pulse-type populations of *Lu. longipalpis* [16]. Considering that *Lu. cruzi* males produce Burst-type song, it is tempting to speculate that *para* might be an important genetic determinant of song type (Burst vs. Pulse) between the two groups of *Lu. longipalpis* populations. Alternatively, *para* and *per* might be linked, with different levels of linkage disequilibrium and/or ancestral polymorphisms, to other loci associated with the reproductive isolation between the Brazilian sibling species.

The *D. melanogaster* courtship song genes are involved in a number of different molecular functions (reviewed in [12]). The three song genes used so far to study the *Lu. longipalpis* complex, *para, cac* and *per* encode, respectively, a voltage-gated sodium channel, a voltage-gated calcium channel, and a transcriptional repressor primarily involved in the circadian clock. It is possible that future RNA interference experiments (e.g. [57]) will help to confirm the potential role of these and other song genes in controlling copulation song differences among *Lu. longipalpis* sibling species. In addition, playback experiments (e.g. [7]–[9], [58]) should also be carried out to directly infer whether copulation songs are involved in mate choice and reproductive isolation.

Finally, our *para* data also confirm existence of three localities (Sobral, Jaiba and Estrela) where pairs of species carrying different spot phenotypes and producing either Burst-type or Pulse-type songs occur in sympatry [16]. The existence of fixed differences in *para*, allowing easy genotyping of females of the different species, will be particularly useful in these three localities to investigate whether the Burst-type and Pulse-type song females show any differences in other aspects of behavior when they occur sympatrically. The study of such phenotypic differences among closely related or incipient vector species is necessary because of the evolutionary and epidemiological implications of traits such as host or habitat preferences that have potential roles in ecological speciation [59] and/or in vector capacity [20].

## Supporting Information

Figure S1 **Alignment of the** ***paralytic*** **gene whole fragment.** Intron sequence is highlighted in grey and non-recombinant block used to construct the haplotype network is highlighted in yellow. Dots indicate the same nucleotide and dashes indicate gaps.
(DOC)

Table S1 **Distribution of the 40 haplotypes found among** ***Lu. longipalpis*** **and** ***Lu. cruzi*** **samples, segregating sites within a 251 bp non-recombinant fragment and number of sequences represented in each sample.**
(DOC)
