# Supplementary material for: Fixed Differences in the paralytic Gene Define Two Lineages within the Lutzomyia longipalpis Complex Producing Different Types of Courtship Songs
Source: PLoS One. 2012 Sep 7;7(9):e44323. doi: 10.1371/journal.pone.0044323 (PMC3436889; doi:10.1371/journal.pone.0044323)
Supplement: Figure S1 — Alignment of the paralytic gene whole fragment. Intron sequence is highlighted in grey and non-recombinant block used to construct the haplotype network is highlighted in yellow. Dots indicate the same nucleotide and dashes indicate gaps. (DOC) [file pone.0044323.s001.doc]

**Figure S1: Alignment of the *paralytic* gene whole fragment.** Intron sequence is highlighted in grey and non-recombinant block used to construct the haplotype network is highlighted in yellow. Dots indicate the same nucleotide and dashes indicate gaps.

111111111111111111111111111111

111111111122222222223333333333444444444455555555556666666666777777777788888888889999999999000000000011111111112222222222

123456789012345678901234567890123456789012345678901234567890123456789012345678901234567890123456789012345678901234567890123456789

lap_14B ATGATAGTATTCCGTGTGCTGTGCGGCGAATGGATTGAGTCAATGTGGGATTGCATGCTGGTGGGAGATGTATCATGCATTCCTTTCTTCTTGGCAACAGTAGTAATTGGGAATTTAGTCGTGAGTATG

lap_14A .................................................................................................................................

lap_15A .................................................................................................................................

lap_15AA .................................................................................................................................

lap_13A .................................................................................................................................

lap_13AA .................................................................................................................................

lap_17A .................................................................................................................................

lap_17AA .................................................................................................................................

lap_18A .................................................................................................................................

lap_18AA .................................................................................................................................

lap_16A .................................................................................................................................

lap_16AA .................................................................................................................................

lap_2B .................................................................................................................................

lap_2A .................................................................................................................................

lap_5B .................................................................................................................................

lap_5A .................................................................................................................................

lap_12B .................................................................................................................................

lap_12A .................................................................................................................................

lap_11B .................................................................................................................................

lap_11A .................................................................................................................................

lap_9B .................................................................................................................................

lap_9A .................................................................................................................................

lap_8A .................................................................................................................................

lap_8AA .................................................................................................................................

lap_6A .................................................................................................................................

lap_6AA .................................................................................................................................

lap_10B .................................................................................................................................

lap_10A .................................................................................................................................

jac_3A .................................................................................................................................

jac_3AA .................................................................................................................................

jac_14A .................................................................................................................................

jac_14AA .................................................................................................................................

jac_10B .................................................................................................................................

jac_10A .................................................................................................................................

jac_1B .................................................................................................................................

jac_1A ..................T..............................................................................................................

jac_9A .................................................................................................................................

jac_9AA .................................................................................................................................

jac_7B .................................................................................................................................

jac_7A .................................................................................................................................

jac_4A ..................T..............................................................................................................

jac_4AA ..................T..............................................................................................................

jac_5B ..................T..............................................................................................................

jac_5A ..................T..............................................................................................................

jac_2B .................................................................................................................................

jac_2A .................................................................................................................................

jac_13B .................................................................................................................................

jac_13A .................................................................................................................................

jac_11A ..................T..............................................................................................................

jac_11B .................................................................................................................................

sob1S_6A .................................................................................................................................

sob1S_6B .................................................................................................................................

sob1S_9A .................................................................................................................................

sob1S_9AA .................................................................................................................................

sob1S_1B .................................................................................................................................

sob1S_1A .................................................................................................................................

sob1S_8B .................................................................................................................................

sob1S_8A .................................................................................................................................

sob1S_2A .................................................................................................................................

sob1S_2B .................................................................................................................................

sob1S_18B .................................................................................................................................

sob1S_18A .................................................................................................................................

sob1S_17B .................................................................................................................................

sob1S_17A .................................................................................................................................

sob1S_16B .................................................................................................................................

sob1S_16A .................................................................................................................................

sob1S_13A .................................................................................................................................

sob1S_13AA .................................................................................................................................

sob1S_5B .................................................................................................................................

sob1S_5A .................................................................................................................................

sob1S_4A .................................................................................................................................

sob1S_4AA .................................................................................................................................

sob1S_15B .................................................................................................................................

sob1S_15A .................................................................................................................................

sob1S_14A .................................................................................................................................

sob1S_14AA .................................................................................................................................

sob1S_12B .................................................................................................................................

sob1S_12A .................................................................................................................................

sob1S_10B .................................................................................................................................

sob1S_10A .................................................................................................................................

sob1S_11A .................................................................................................................................

sob1S_11AA .................................................................................................................................

est1S_7A .................................................................................................................................

est1S_7AA .................................................................................................................................

est1S_8A ..................T..............................................................................................................

est1S_8B ..................T..............................................................................................................

est1S_4A .................................................................................................................................

est1S_4AA .................................................................................................................................

est1S_6A .................................................................................................................................

est1S_6AA .................................................................................................................................

est1S_3A .................................................................................................................................

est1S_3AA .................................................................................................................................

est1S_9A .................................................................................................................................

est1S_9AA .................................................................................................................................

est1S_5B .................................................................................................................................

est1S_5A .................................................................................................................................

est1S_12A .................................................................................................................................

est1S_12AA .................................................................................................................................

est1S_11A .................................................................................................................................

est1S_11AA .................................................................................................................................

est1S_10A .................................................................................................................................

est1S_10AA .................................................................................................................................

est1S_s9A .................................................................................................................................

est1S_s9AA .................................................................................................................................

jai1S_s1A .................................................................................................................................

jai1S_s1B .................................................................................................................................

jai1S_s2A .................................................................................................................................

jai1S_s2B .................................................................................................................................

jai1S_s3A .................................................................................................................................

jai1S_s3B .................................................................................................................................

jai1S_s4A .................................................................................................................................

jai1S_s4B .................................................................................................................................

jai1S_1A .................................................................................................................................

jai1S_1B .................................................................................................................................

jai1S_2A .................................................................................................................................

jai1S_2B .................................................................................................................................

jai1S_3A .................................................................................................................................

jai1S_3AA .................................................................................................................................

jai1S_4A .................................................................................................................................

jai1S_4B .................................................................................................................................

jai1S_5A .................................................................................................................................

jai1S_5AA .................................................................................................................................

jai1S_6A .................................................................................................................................

jai1S_6AA .................................................................................................................................

jai1S_7A .................................................................................................................................

jai1S_7B .................................................................................................................................

jai1S_8A .................................................................................................................................

jai1S_8AA .................................................................................................................................

ter_1A .................................................................................................................................

ter_1B .................................................................................................................................

ter_2A .................................................................................................................................

ter_2B .................................................................................................................................

ter_3A .................................................................................................................................

ter_3B .................................................................................................................................

ter_4A .................................................................................................................................

ter_4AA .................................................................................................................................

ter_5A .................................................................................................................................

ter_5B .................................................................................................................................

ter_6A .................................................................................................................................

ter_6B .................................................................................................................................

ter_7A .................................................................................................................................

ter_7B .................................................................................................................................

ter_8A .................................................................................................................................

ter_8B .................................................................................................................................

ter_9A .................................................................................................................................

ter_9B .................................................................................................................................

ter_10A .................................................................................................................................

ter_10B .................................................................................................................................

ter_11A .................................................................................................................................

ter_11AA .................................................................................................................................

ter_12A .................................................................................................................................

ter_12AA .................................................................................................................................

pan_4B ..................T..............................................................................................................

pan_4A ..................T..............................................................................................................

pan_8A ..................T..............................................................................................................

pan_8AA ..................T..............................................................................................................

pan_2B ..............C...T..............................................................................................................

pan_2A ..................T..............................................................................................................

pan_18B ..................T..............................................................................................................

pan_18A ..................T..............................................................................................................

pan_14A ..................T..............................................................................................................

pan_14AA ..................T..............................................................................................................

pan_9B ..................T..............................................................................................................

pan_9A ..................T..............................................................................................................

pan_7A ..................T..............................................................................................................

pan_7AA ..................T..............................................................................................................

pan_6B ..................T..............................................................................................................

pan_6A ..................T..............................................................................................................

pan_17B ..................T..............................................................................................................

pan_17A ..................T..............................................................................................................

pan_16B ..................T..............................................................................................................

pan_16A ..................T..............................................................................................................

pan_15B ..................T..............................................................................................................

pan_15A ..................T..............................................................................................................

pan_12B ..................T..............................................................................................................

pan_12A ..................T..............................................................................................................

pan_11B ..................T..............................................................................................................

pan_11A ..................T..............................................................................................................

pan_21B ..................T..............................................................................................................

pan_21A ..................T..............................................................................................................

pan_19B ..................T..............................................................................................................

pan_19A ..................T..............................................................................................................

pan_13B ..................T..............................................................................................................

pan_13A ..................T..............................................................................................................

nat_7B ..................T..............................................................................................................

nat_7A ..................T..............................................................................................................

nat_6B ..................T..............................................................................................................

nat_6A ..................T..............................................................................................................

nat_5B ..................T..............................................................................................................

nat_5A ..................T..............................................................................................................

nat_4B ..................T..............................................................................................................

nat_4A ..................T..............................................................................................................

nat_3B ..................T..............................................................................................................

nat_3A ..................T..............................................................................................................

nat_2B ..................T..............................................................................................................

nat_2A ..................T..............................................................................................................

nat_11B ..................T..............................................................................................................

nat_11A ..................T.........................................................................A....................................

nat_10B ..................T..............................................................................................................

nat_10A ..................T..............................................................................................................

nat_9B ..................T..............................................................................................................

nat_9A ..................T..............................................................................................................

nat_8B ..................T..............................................................................................................

nat_8A ..................T..............................................................................................................

nat_1B ..................T..............................................................................................................

nat_1A ..................T..............................................................................................................

nat_12B ..................T..............................................................................................................

nat_12A ..................T..............................................................................................................

sob2S_6A ..................T..............................................................................................................

sob2S_6AA ..................T..............................................................................................................

sob2S_11A ..................T..............................................................................................................

sob2S_11AA ..................T..............................................................................................................

sob2S_5A ..................T..............................................................................................................

sob2S_5AA ..................T..............................................................................................................

sob2S_4B ..................T..............................................................................................................

sob2S_4A ..................T..............................................................................................................

sob2S_1A ..................T..............................................................................................................

sob2S_1AA ..................T..............................................................................................................

sob2S_18B ..................T..............................................................................................................

sob2S_18A ..................T..............................................................................................................

sob2S_17B ..................T..............................................................................................G...............

sob2S_17A ..................T..............................................................................................................

sob2S_15A ..................T..............................................................................................................

sob2S_15AA ..................T..............................................................................................................

sob2S_13A ..................T..............................................................................................................

sob2S_13AA ..................T..............................................................................................................

sob2S_9B ..................T..............................................................................................................

sob2S_9A ..................T..............................................................................................................

sob2S_8B ..................T..............................................................................................................

sob2S_8A ..................T..............................................................................................................

sob2S_7B ..................T..............................................................................................................

sob2S_7A ..................T..............................................................................................................

sob2S_16B ..................T..............................................................................................................

sob2S_16A ..................T..............................................................................................................

sob2S_14B ..................T..............................................................................................................

sob2S_14A ..................T..............................................................................................................

jai2S_s9B ..................T..............................................................................................................

jai2S_s8A ..................T..............................................................................................................

jai2S_2A ..................T..............................................................................................................

jai2S_2AA ..................T..............................................................................................................

jai2S_s12A ..................T..............................................................................................................

jai2S_s12AA ..................T..............................................................................................................

jai2S_s3B ..................T..............................................................................................................

jai2S_s4B ..................T..............................................................................................................

jai2S_s5B ..................T..............................................................................................................

jai2S_s6A ..................T..............................................................................................................

jai2S_s6AA ..................T..............................................................................................................

jai2S_s4A ..................T..............................................................................................................

jai2S_s7A ..................T..............................................................................................................

jai2S_s10A ..................T..............................................................................................................

jai2S_s10B ..................T..............................................................................................................

jai2S_s11B ..................T..............................................................................................................

jai2S_s11A ..................T..............................................................................................................

jai2S_1B ..................T..............................................................................................................

jai2S_1A ..................T..............................................................................................................

jai2S_s8B ..................T..............................................................................................................

jai2S_s5A ..................T..............................................................................................................

jai2S_s7B ..................T..............................................................................................................

jai2S_s9A ..................T..............................................................................................................

jai2S_s3A ..................T............................................................................................G.................

est2S_26A ..................T..............................................................................................................

est2S_25A ..................T..............................................................................................................

est2S_28A ..................T..............................................................................................................

est2S_27B ..................T..............................................................................................................

est2S_23A ..................T..............................................................................................................

est2S_22A ..................T..............................................................................................................

est2S_32A ..................T..............................................................................................................

est2S_32AA ..................T..............................................................................................................

est2S_27A ..................T..............................................................................................................

est2S_24A ..................T..............................................................................................................

est2S_29A ..................T..............................................................................................................

est2S_23B ..................T..............................................................................................................

est2S_25B ..................T..............................................................................................................

est2S_31A ..................T..............................................................................................................

est2S_29B ..................T..............................................................................................................

est2S_28B ..................T..............................................................................................................

est2S_24B ..................T..............................................................................................................

est2S_22B ..................T..............................................................................................................

est2S_31B ..................T..............................................................................................................

est2S_26B ..................T..............................................................................................................

est2S_s1A ..................T..............................................................................................................

est2S_s1B ..................T..............................................................................................................

est2S_s3A ..................T..............................................................................................................

est2S_s3B ..................T..............................................................................................................

est2S_s4A ..................T..............................................................................................................

est2S_s4B ..................T..............................................................................................................

est2S_s5A ..................T..............................................................................................................

est2S_s5AA ..................T..............................................................................................................

est2S_s6A ..................T..............................................................................................................

est2S_s6B ..................T..............................................................................................................

est2S_s7A ..................T..............................................................................................................

est2S_s7AA ..................T..............................................................................................................

mar_1A ..................T..............................................................................................................

mar_1AA ..................T..............................................................................................................

mar2A ..................T..............................................................................................................

mar_2B ..................T..............................................................................................................

mar_11A ..................T..............................................................................................................

mar_11B ..................T..............................................................................................................

cruzi_1A ..................T..............................................................................................................

cruzi_1B ..................T..............................................................................................................

cruzi_2A ..................T..............................................................................................................

cruzi_2B ..................T..............................................................................................................

cruzi_3A ..................T..............................................................................................................

cruzi_3AA ..................T..............................................................................................................

cruzi_4A ..................T..............................................................................................................

cruzi_4B ..................T..............................................................................................................

cruzi_5A ..................T..............................................................................................................

cruzi_5AA ..................T..............................................................................................................

cruzi_6A ..................T..............................................................................................................

cruzi_6B ..................T..............................................................................................................

cruzi_7A ..................T..............................................................................................................

cruzi_7AA ..................T..............................................................................................................

cruzi_8A ..................T..............................................................................................................

cruzi_8AA ..................T..............................................................................................................

cruzi_9A ..................T..............................................................................................................

cruzi_9B ..................T..............................................................................................................

cruzi_10A ..................T..............................................................................................................

cruzi_10B ..................T..............................................................................................................

cruzi_11A ..................T..............................................................................................................

cruzi_11B ..................T..................G...........................................................................................

cruzi_12A ..................T..............................................................................................................

cruzi_12B ..................T..............................................................................................................

pseudo_14A .................................................................................................................................

pseudo_14AA .................................................................................................................................

pseudo_25A .................................................................................................................................

pseudo_25AA .................................................................................................................................

111111111111111111111111111111111111111111111111111111111111111111111122222222222222222222222222222222222222222222222222222222222

333333333344444444445555555555666666666677777777778888888888999999999900000000001111111111222222222233333333334444444444555555555

012345678901234567890123456789012345678901234567890123456789012345678901234567890123456789012345678901234567890123456789012345678

lap_14B AAATTGAATATGATATTCAACACACCCTCGTTGGACGGGGGG-TGGAATGGTGTATATATTTGTGGATATATGGGGCGGAAATGAGTCCCATTTGCATCGATTGAGTGTTGTGCGACCACTAAATGTTG

lap_14A ..........................................-......................................................................................

lap_15A ..........................................-......................................................................................

lap_15AA ..........................................-......................................................................................

lap_13A ..........................................-......................................................................................

lap_13AA ..........................................-......................................................................................

lap_17A ..........................................-...................A..................................................................

lap_17AA ..........................................-...................A..................................................................

lap_18A ..........................................-...................A..................................................................

lap_18AA ..........................................-...................A..................................................................

lap_16A ..........................................-...................A..................................................................

lap_16AA ..........................................-...................A..................................................................

lap_2B ..........................................-......................................................................................

lap_2A ..........................................-......................................................................................

lap_5B ..........................................-......................................................................................

lap_5A ..........................................-......................................................................................

lap_12B ..........................................-......................................................................................

lap_12A ..........................................-......................................................................................

lap_11B ......................................A...-......................................................................................

lap_11A ..........................................-...................A..................................................................

lap_9B ..........................................-...................A..................................................................

lap_9A ..........................................-......................................................................................

lap_8A ..........................................-...................A..................................................................

lap_8AA ..........................................-...................A..................................................................

lap_6A ..........................................-...................A..................................................................

lap_6AA ..........................................-...................A..................................................................

lap_10B ..........................................-...................A..................................................................

lap_10A ..........................................-...................A..................................................................

jac_3A ..........................................-.......T..--..........................................................................

jac_3AA ..........................................-.......T..--..........................................................................

jac_14A ..........................................-.A.....T..--..........................................................................

jac_14AA ..........................................-.A.....T..--..........................................................................

jac_10B ..........................................-.......T..--..........................................................................

jac_10A ..........................................-.......T..--.........................................................A................

jac_1B ..........................................-.A.....T..--..........................................................................

jac_1A ..........................................-......................................................................................

jac_9A ..........................................-......................................................................................

jac_9AA ..........................................-......................................................................................

jac_7B ..........................................-......................................................................................

jac_7A ..........................................-............................................................A.........................

jac_4A ..........................................-......................................................................................

jac_4AA ..........................................-......................................................................................

jac_5B .............C............................-......................................................................................

jac_5A ..........................................-......................................................................................

jac_2B ..........................G...............-......................................................................................

jac_2A ..........................................-......................................................................................

jac_13B ..........................................-......................................................................................

jac_13A ..................G.......................-.......T..--..........................................................................

jac_11A ..........................................-......................................................................................

jac_11B ..........................................-.......T..--..........................................................................

sob1S_6A ..........................................-......................................................................................

sob1S_6B ..........................................-......................................................................................

sob1S_9A ..........................................-.......T..--..........................................................................

sob1S_9AA ..........................................-.......T..--..........................................................................

sob1S_1B ..........................................-......................................................................................

sob1S_1A ..........................................-.............................................T........................................

sob1S_8B ..........................................-......................................................................................

sob1S_8A ..........................................-......................................................................................

sob1S_2A ..........................................-......................................................................................

sob1S_2B ..........................................-.........A............................................................................

sob1S_18B ..........................................-......................................................................................

sob1S_18A ..........................................-.........A............................................................................

sob1S_17B ..........................................-...................A..................................................................

sob1S_17A ..........................................-......................................................A...............................

sob1S_16B ..........................................-............................................................A.........................

sob1S_16A ..........................................-......................................................................................

sob1S_13A ..........................................-......................................................................................

sob1S_13AA ..........................................-......................................................................................

sob1S_5B ..........................................-......................................................................................

sob1S_5A ..........................................-......................................................................................

sob1S_4A ..........................................-......................................................................................

sob1S_4AA ..........................................-......................................................................................

sob1S_15B ..........................................-............................................................A.........................

sob1S_15A ..........................................-......................................................................................

sob1S_14A ..........................................-......................................................................................

sob1S_14AA ..........................................-......................................................................................

sob1S_12B ..........................................-......................................................................................

sob1S_12A ..........................................-...........................................C..........................................

sob1S_10B ..........................................-......................................................................................

sob1S_10A ...................................T......-......................................................................................

sob1S_11A ..........................................-......................................................................................

sob1S_11AA ..........................................-......................................................................................

est1S_7A ..........................................-.......T..--..........................................................................

est1S_7AA ..........................................-.......T..--..........................................................................

est1S_8A ..........................................-.......T..--..........................................................T........T......

est1S_8B ..........................................-.......T..--..........................................................T........T......

est1S_4A ..........................................-.......T..--..........................................................................

est1S_4AA ..........................................-.......T..--..........................................................................

est1S_6A ..........................................-.......T..--..........................................................................

est1S_6AA ..........................................-.......T..--..........................................................................

est1S_3A ..........................................-.......T..--..........................................................................

est1S_3AA ..........................................-.......T..--..........................................................................

est1S_9A ..........................................-.......T..--..........................................................................

est1S_9AA ..........................................-.......T..--..........................................................................

est1S_5B ..........................................-.......T..--..........................................................................

est1S_5A ..........................................-.......T..--..........................................................................

est1S_12A ..........................................-.......T..--..........................................................................

est1S_12AA ..........................................-.......T..--..........................................................................

est1S_11A ..........................................-.......T..--..........................................................................

est1S_11AA ..........................................-.......T..--..........................................................................

est1S_10A ..........................................-.......T..--..........................................................................

est1S_10AA ..........................................-.......T..--..........................................................................

est1S_s9A ..........................................-.......T..--..........................................................................

est1S_s9AA ..........................................-.......T..--..........................................................................

jai1S_s1A ..........................................-......................................................................................

jai1S_s1B ..........................................-......................................................................................

jai1S_s2A ..........................................-......................................................................................

jai1S_s2B ..........................................-......................................................................................

jai1S_s3A ..........................................-......................................................................................

jai1S_s3B ..........................................-......................................................................................

jai1S_s4A ..........................................-......................................................................................

jai1S_s4B ..........................................-......................................................................................

jai1S_1A ..........................................-...................A..................................................................

jai1S_1B ..........................................-................................A.....................................................

jai1S_2A ..........................................G...................A..................................................................

jai1S_2B ..........................................-......................................................................................

jai1S_3A ..........................................-......................................................................................

jai1S_3AA ..........................................-......................................................................................

jai1S_4A .........................................--......................................................................................

jai1S_4B ..........................................-......................................................................................

jai1S_5A ..........................................-......................................................................................

jai1S_5AA ..........................................-......................................................................................

jai1S_6A ..........................................-......................................................................................

jai1S_6AA ..........................................-......................................................................................

jai1S_7A ..........................................-......................................................................................

jai1S_7B ..........................................-......................................................................................

jai1S_8A ..........................................-......................................................................................

jai1S_8AA ..........................................-......................................................................................

ter_1A ..........................................-......................................................................................

ter_1B ..........................................-......................................................................................

ter_2A ..........................................-......................................................................................

ter_2B ..........................................-.......T.......--.....................................................................

ter_3A ..........................................-......................................................................................

ter_3B ...................................T......-......................................................................................

ter_4A ..........................................-......................................................................................

ter_4AA ..........................................-......................................................................................

ter_5A ..........................................-......................................................................................

ter_5B ..........................................-......................................................A...............................

ter_6A ..........................................-......................................................................................

ter_6B ..........................................-......................................................................................

ter_7A ..........................................-......................................................................................

ter_7B ........................T.................-......................................................................................

ter_8A ..........................................-............................................................................T.........

ter_8B ..........................................-......................................................................................

ter_9A ..........................................-......................................................................................

ter_9B ..........................................-......................................................................................

ter_10A ..........................................-......................................................................................

ter_10B ..........................................-......................................................................................

ter_11A ..........................................-......................................................................................

ter_11AA ..........................................-......................................................................................

ter_12A ..........................................-......................................................................................

ter_12AA ..........................................-......................................................................................

pan_4B ..........................................-.......T..--..........................................................T........T......

pan_4A ..........................................-.......T..--..........................................................T........T......

pan_8A ..........................................-.......T..--.....................................................C....T........T......

pan_8AA ..........................................-.......T..--.....................................................C....T........T......

pan_2B ..........................................-.......T..--..........................................................T........T......

pan_2A ..........................................-.......T..--..........................................................T........T......

pan_18B ..........................................-.......T..--..........................................................T........T......

pan_18A ..........................................-.......T..--.....................................................C....T........T......

pan_14A ..........................................-.......T..--..........................................................T........T......

pan_14AA ..........................................-.......T..--..........................................................T........T......

pan_9B ..........................................-.......T..--..........................................................T........T......

pan_9A ..........................................-.......T..--..........................................................T........T......

pan_7A ..........................................-.......T..--..........................................................T........T......

pan_7AA ..........................................-.......T..--..........................................................T........T......

pan_6B ..........................................-.......T..--.....................................................C....T........T......

pan_6A ..........................................-.......T..--.....................................................C....T........T......

pan_17B ..........................................-.......T..--...................................................C......T........T......

pan_17A ..........................................-.......T..--..........................................................T........T......

pan_16B ..........................................-.......T..--..........................................................T........T......

pan_16A ..........................................-.......T..--..........................................................T........T......

pan_15B ..........................................-.......T..--..........................................................T........T......

pan_15A ..........................................-.......T..--..........................................................T........T......

pan_12B ..........................................-.......T..--..........................................................T........T......

pan_12A ..........................................-.......T..--..........................................................T........T......

pan_11B ..........................................-.......T..--..........................................................T........T......

pan_11A ..........................................-.......T..--.....................................................C....TA.......T......

pan_21B ..........................................-.......T..--..........................................................T........T......

pan_21A ..........................................-.......T..--..........................................................T........T......

pan_19B ..........................................-.......T..--..........................................................T........T......

pan_19A ..........................................-.......T..--..........................................................T........T......

pan_13B ..........................................-.......T..--..........................................................T........T......

pan_13A ..........................................-.......T..--..........................................................T........T......

nat_7B ..........................................-.......T..--..........................................................T........T......

nat_7A ..........................................-.......T..--..........................................................T........T......

nat_6B ..........................................-.......T..--......................................................C...T........T......

nat_6A ..........................................-.......T..--..........................................................T........T......

nat_5B ..........................................-.......T..--.....................................................C....T........T......

nat_5A ..........................................-.......T..--..........................................................T........T......

nat_4B ..........................................-.......T..--.....................................................C....T........T......

nat_4A ..........................................-.......T..--..........................................................T........T......

nat_3B ..........................................-.......T..--..........................................................T........T......

nat_3A ..........................................-.......T..--..........................................................T........T......

nat_2B ..........................................-.......T..--..........................................................T........T......

nat_2A ..........................................-.......T..--..........................................................T........T......

nat_11B ..........................................-.......T..--..........................................................T........T......

nat_11A ..........................................-.......T..--..........................................................T........T......

nat_10B ..........................................-.......T..--..........................................................T........T......

nat_10A ..........................................-.......T..--..........................................................T........T......

nat_9B ..........................................-.......T..--.....................................................C....T........T......

nat_9A ..........................................-.......T..--..................................................C.......T........T......

nat_8B ..........................................-.......T..--..........................................................T........T......

nat_8A ..........................................-.......T..--.....................G....................................T........T......

nat_1B ..........................................-.......T..--..........................................................T........T......

nat_1A ..........................................-.......T..--..........................................................T........T......

nat_12B ..........................................-.......T..--..........................................................T........T......

nat_12A ..........................................-.......T..--..........................................................T........T......

sob2S_6A ..........................................-.......T..--..........................................................T........T......

sob2S_6AA ..........................................-.......T..--..........................................................T........T......

sob2S_11A ..........................................-.......T..--............C.............................................T........T......

sob2S_11AA ..........................................-.......T..--............C.............................................T........T......

sob2S_5A ..........................................-.......T..--..........................................................T........T......

sob2S_5AA ..........................................-.......T..--..........................................................T........T......

sob2S_4B ..........................................-.......T..--..........................................................T........T......

sob2S_4A ..........................................-.......T..--............C.............................................T........T......

sob2S_1A ..........................................-.......T..--..........................................................T........T......

sob2S_1AA ..........................................-.......T..--..........................................................T........T......

sob2S_18B ..........................................-.......T..--..........................................................T........T......

sob2S_18A ..........................................-.......T..--..........................................................T........T......

sob2S_17B ..........................................-.......T..--..........................................................T........T......

sob2S_17A ..........................................-.......T..--..........................................................T........T......

sob2S_15A ..........................................-.......T..--..........................................................T........T......

sob2S_15AA ..........................................-.......T..--..........................................................T........T......

sob2S_13A ..........................................-.......T..--..........................................................T........T......

sob2S_13AA ..........................................-.......T..--..........................................................T........T......

sob2S_9B ..........................................-.......T..--..........................................................T........T......

sob2S_9A ..........................................-.......T..--..........................................................T........T......

sob2S_8B ..........................................-.......T..--..........................................................T........T......

sob2S_8A ..........................................-.......T..--..........................................................T........T......

sob2S_7B ..........................................-.......T..--..........................................................T........T......

sob2S_7A ..........................................-.......T..--..........................................................T........T......

sob2S_16B ..........................................-.......T..--..........................................................T........T......

sob2S_16A ..........................................-.......T..--..........................................................T........T......

sob2S_14B ..........................................-.......T..--..........................................................T........T......

sob2S_14A ..........................................-.......T..--..........................................................T........T......

jai2S_s9B ..........................................-.......T..--..........................................................T........T......

jai2S_s8A ..........................................-.......T..--..........................................................T........T......

jai2S_2A ..........................................-.......T..--..........................................................T........T......

jai2S_2AA ..........................................-.......T..--..........................................................T........T......

jai2S_s12A ..........................................-.......T..--..........................................................T........T......

jai2S_s12AA ..........................................-.......T..--..........................................................T........T......

jai2S_s3B ..........................................-.......T..--..........................................................T........T......

jai2S_s4B ..........................................-.......T..--..........................................................T........T......

jai2S_s5B ..........................................-.......T..--..........................................................T........T......

jai2S_s6A ..........................................-.......T..--..........................................................T........T......

jai2S_s6AA ..........................................-.......T..--..........................................................T........T......

jai2S_s4A ..........................................-.......T..--.....................................................C....T........T......

jai2S_s7A ..........................................-.......T..--.....................................................C....T........T......

jai2S_s10A ..........................................-.......T..--.....................................................C....T........T......

jai2S_s10B .....................................A....-.......T..--..........................................................T........T......

jai2S_s11B ..........................................-.......T..--..........................................................T........T......

jai2S_s11A ..........................................-.......T..--..........................................................T........T......

jai2S_1B ..........................................-.......T..--..........................................................T........T......

jai2S_1A ..........................................-.......T..--..........................................................T........T......

jai2S_s8B ..........................................-.......T..--..........................................................T........T......

jai2S_s5A ..........................................-.......T..--.....................................................C....T........T......

jai2S_s7B ..........................................-.......T..--..........................................................T........T......

jai2S_s9A ..........................................-.......T..--..........................................................T.C......T......

jai2S_s3A ..........................................-.......T..--..........................................................T........T...C..

est2S_26A ..........................................-.......T..--..........................................................T........T......

est2S_25A ..........................................-.......T..--..........................................................T........T......

est2S_28A ..........................................-.......T..--..........................................................T........T......

est2S_27B ..........................................-.......T..--..........................................................T........T......

est2S_23A ..........................................-.......T..--..........................................................T........T......

est2S_22A ..........................................-.......T..--..........................................................T........T......

est2S_32A ..........................................-.......T..--..........................................................T........T......

est2S_32AA ..........................................-.......T..--..........................................................T........T......

est2S_27A ..........................................-.......T..--..........................................................T........T......

est2S_24A ..........................................-.......T..--..........................................................T........T......

est2S_29A ..........................................-.......T..--..........................................................T........T......

est2S_23B ..........................................-.......T..--..........................................................T........T......

est2S_25B ..........................................-.......T..--..........................................................T........T......

est2S_31A ..........................................-.......T..--..........................................................T........T......

est2S_29B ..........................................-.......T..--..........................................................T........T......

est2S_28B ..........................................-.......T..--.....................................................C....T........T......

est2S_24B ..........................................-.......T..--..........................................................T........T......

est2S_22B ..........................................-.......T..--............C.............................................T........T......

est2S_31B ..........................................-.......T..--..........................................................T........T......

est2S_26B ..........................................-.......T..--..........................................................T........T......

est2S_s1A ..........................................-.......T..--..........................................................T........T......

est2S_s1B ..........................................-.......T..--.....................................................C....T........T......

est2S_s3A ..........................................-.......T..--..........................................................T........T......

est2S_s3B ..........................................-.......T..--..........................................................T........T......

est2S_s4A ..........................................-.......T..--..........................................................T........T......

est2S_s4B ..........................................-.......T..--..........................................................T........T......

est2S_s5A ..........................................-.......T..--..........................................................T........T......

est2S_s5AA ..........................................-.......T..--..........................................................T........T......

est2S_s6A ..........................................-.......T..--............C.............................................T........T......

est2S_s6B ..........................................-.......T..--..........................................................T........T......

est2S_s7A ..........................................-.......T..--..........................................................T........T......

est2S_s7AA ..........................................-.......T..--..........................................................T........T......

mar_1A ..........................................-.......T..--..........................................................T........T......

mar_1AA ..........................................-.......T..--..........................................................T........T......

mar2A ..........................................-.......T..--..........................................................T........T......

mar_2B ..........................................-.......T..--..........................................................T........T......

mar_11A ..........................................-.......T..--..........................................................T........T......

mar_11B ..........................................-.......T..--..........................................................T........T......

cruzi_1A ..........................................-.......T..--..........................................................T........T......

cruzi_1B ..........................................-.......T..--................................................--------..T........T......

cruzi_2A .......................G..................-.......T..--................................................--------..T........T......

cruzi_2B ..........................................-.......T..--................................................--------..T........T......

cruzi_3A ..........................................-.......T..--................................................--------..T........T......

cruzi_3AA ..........................................-.......T..--................................................--------..T........T......

cruzi_4A ..........................................-.......T..--................................................--------..T........T......

cruzi_4B ..........................................-.......T..--................................................--------..T........T......

cruzi_5A ..........................................-.......T..--................................................--------..T........T......

cruzi_5AA ..........................................-.......T..--................................................--------..T........T......

cruzi_6A ..........................................-.......T..--................................................--------..T........T......

cruzi_6B ..........................................-.......T..--................................................--------..T........T......

cruzi_7A ..........................................-.......T..--................................................--------..T........T......

cruzi_7AA ..........................................-.......T..--................................................--------..T........T......

cruzi_8A ..........................................-.......T..--................................................--------..T........T......

cruzi_8AA ..........................................-.......T..--................................................--------..T........T......

cruzi_9A ..........................................-.......T..--................................................--------..T........T......

cruzi_9B ..........................................-.......T..--................................................--------..T........T......

cruzi_10A ..........................................-.......T..--................................................--------..T........T......

cruzi_10B ..........................................-.......T..--................................................--------..T........T......

cruzi_11A ..........................................-.......T..--................................................--------..T........T......

cruzi_11B ..........................................-.......T..--................................................--------..T........T......

cruzi_12A ..........................................-.......T..--................................................--------..T........T......

cruzi_12B ..........................................-.......T..--................................................--------..T........T......

pseudo_14A .....................................AA...-.......T..--..................A.......................................T........T......

pseudo_14AA .....................................AA...-.......T..--..................A.......................................T........T......

pseudo_25A .....................................AA...-.......T..--..................A.......................................T........T......

pseudo_25AA .....................................AA...-.......T..--..................A.......................................T........T......

2222222222222222222222222222222222222222233333333333333333333333333333333333333333333333333333333333333333333333333333333333333

5666666666677777777778888888888999999999900000000001111111111222222222233333333334444444444555555555566666666667777777777888888

9012345678901234567890123456789012345678901234567890123456789012345678901234567890123456789012345678901234567890123456789012345

lap_14B TGTC-CAT--ACACAGCA-TAAAATGT-TACAAAATTAAATG--CACTAATAATGACTTTTTTTT----GAATCCCTTTTCTAGGTTCTCAATCTTTTCTTAGCCTTGCTTTTGAGCAATTTCGGAT

lap_14A ....-...--........-........A..............--.....................----..........................................................

lap_15A ....-...--........-........A..............--.....................----..........................................................

lap_15AA ....-...--........-........A..............--.....................----..........................................................

lap_13A ....-...--........-........A..............--.....................----..........................................................

lap_13AA ....-...--........-........A..............--.....................----..........................................................

lap_17A ....-...--........-........A..............--.....................TT--..........................................................

lap_17AA ....-...--........-........A..............--.....................TT--..........................................................

lap_18A ....-...--........-........A..............--.....................TT--..........................................................

lap_18AA ....-...--........-........A..............--.....................TT--..........................................................

lap_16A ....-...--........-........A..............--.....................TT--..........................................................

lap_16AA ....-...--........-........A..............--.....................TT--..........................................................

lap_2B ....-...--........-........-..............--.....................----..........................................................

lap_2A ....-...--........-........A..............--.....................----..........................................................

lap_5B ....-...--........-........-..............--.....................----..........................................................

lap_5A ....-...--........-........A..............--.....................T---..........................................................

lap_12B ....-...--........-........-..............--.....................----..........................................................

lap_12A ....-...--........-........A..............--.....................T---..........................................................

lap_11B ....-...--........-........A..............--.....................T---..........................................................

lap_11A ....-...--........-........A..............--.....................T---..........................................................

lap_9B ....-...--........-........A..............--.....................TT--..........................................................

lap_9A ....-...--........-........A..............--.....................TT--..........................................................

lap_8A ....-...--........-........A..............--.....................TTT-..........................................................

lap_8AA ....-...--........-........A..............--.....................TTT-..........................................................

lap_6A ....-...--........-........A..............--.....................TT--..........................................................

lap_6AA ....-...--........-........A..............--.....................TT--..........................................................

lap_10B ....-...--........-........A..............--.....................TT--..........................................................

lap_10A ....-...--........-........A..............--.....................TTT-..........................................................

jac_3A ....-...--.....A..-........-..............--.....................T---..........................................................

jac_3AA ....-...--.....A..-........-..............--.....................T---..........................................................

jac_14A ....-...--........-........-..............--.....................T---..........................................................

jac_14AA ....-...--........-........-..............--.....................T---..........................................................

jac_10B ....-...--........-........-..............--.....................T---..........................................................

jac_10A ....-...--........-........-..............--.....................T---..........................................................

jac_1B ....-...--........-........-..............--.....................T---..........................................................

jac_1A ....-...--........-........-..............--.....................T---..........................................................

jac_9A ....-...--........-........-..............--.....................TT--..........................................................

jac_9AA ....-...--........-........-..............--.....................TT--..........................................................

jac_7B ....-...--........-........-..............--.....................TT--..........................................................

jac_7A ....-...--........-........-..............--.....................TT--..........................................................

jac_4A ....-...--........-........-..............--.....................TT--..........................................................

jac_4AA ....-...--........-........-..............--.....................TT--..........................................................

jac_5B ....-...--........-........-..............--.....................TT--..........................................................

jac_5A ....-...--........-........-..............--.....................TTT-..........................................................

jac_2B ....-...--........-........A..............--.....................T---..........................................................

jac_2A ....-...--........-........-..............--.....................T---..........................................................

jac_13B ....-...--.....A..-........-..............--.....................TT--..........................................................

jac_13A ....-...--........-........-..............--....................-----..........................................................

jac_11A ....-...--........-........-..............--.....................TT--..........................................................

jac_11B ....-...--........-........-..............--.....................----..........................................................

sob1S_6A ....-...--........-A....G..-..............--.....................T---..........................................................

sob1S_6B ....-...--........-........-..............--.....................----..........................................................

sob1S_9A ....-...--........-........-..............--.....................----..........................................................

sob1S_9AA ....-...--........-........-..............--.....................----..........................................................

sob1S_1B ....-...--........-A....G..-..............--.....................T---..........................................................

sob1S_1A ....-...--........-........-..............--.....................----..........................................................

sob1S_8B ....-...--........-........A..............--.....................----..........................................................

sob1S_8A ....-...--........-........A..............--.....................T---..........................................................

sob1S_2A ....-...--........-........A..............--.....................T---..........................................................

sob1S_2B ....-...--........-........A..............--.....................T---..........................................................

sob1S_18B ....-...--........-........A..............--.....................T---..........................................................

sob1S_18A ....-...--........-........A..............--.....................T---..........................................................

sob1S_17B ....-...--........-........A..............--.....................T---..........................................................

sob1S_17A ....-...--...G....-........A..............--.....................T---..........................................................

sob1S_16B ....-...--........-G.......-..............--.....................T---..........................................................

sob1S_16A ....-...--........-........A..............--.....................----......T...................................................

sob1S_13A ....-...--........-........A..............--.....................T---..........................................................

sob1S_13AA ....-...--........-........A..............--.....................T---..........................................................

sob1S_5B ....-...--........-........A..............--.....................T---..........................................................

sob1S_5A ....-...--........-........-..............--.....................TT--..........................................................

sob1S_4A ....-...--........-........A..............--.....................T---..........................................................

sob1S_4AA ....-...--........-........A..............--.....................T---..........................................................

sob1S_15B ....-...--........-G.......-..............--.....................T---..........................................................

sob1S_15A ....-...--........-........A..............--.....................T---..........................................................

sob1S_14A ....-...--........-........A..............--.....................TT--..........................................................

sob1S_14AA ....-...--........-........A..............--.....................TT--..........................................................

sob1S_12B ....-...--........-........A..............--.....................----..........................................................

sob1S_12A ....-...--........-........-..............--.....................T---..........................................................

sob1S_10B ....-...--........-........A..............--.....................TT--......T...................................................

sob1S_10A ....-...--........-........A..............--.....................TT--..........................................................

sob1S_11A ....-...--........-........A..............--.....................----..........................................................

sob1S_11AA ....-...--........-........A..............--.....................----..........................................................

est1S_7A ....-...--........-........-..............--.....................----..........................................................

est1S_7AA ....-...--........-........-..............--.....................----..........................................................

est1S_8A ...--...--........-........-..............--.....................----..........................................................

est1S_8B ...--..G--........-........-..T...........--.....................----..........................................................

est1S_4A ....-...--........-........-..............--.....................----..........................................................

est1S_4AA ....-...--........-........-..............--.....................----..........................................................

est1S_6A ....-...--........-........-..............--.....................----..........................................................

est1S_6AA ....-...--........-........-..............--.....................----..........................................................

est1S_3A ....-...--........-........-..............--.....................----..........................................................

est1S_3AA ....-...--........-........-..............--.....................----..........................................................

est1S_9A ....-...--........-........-..............--.....................T---..........................................................

est1S_9AA ....-...--........-........-..............--.....................T---..........................................................

est1S_5B ....-...--........-........-..............--.....................----..........................................................

est1S_5A ....-...--........-........A..............--.....................T---..........................................................

est1S_12A ....-...--........-........-..............--.....................----..........................................................

est1S_12AA ....-...--........-........-..............--.....................----..........................................................

est1S_11A ....-...--........-........-..............--.....................T---..........................................................

est1S_11AA ....-...--........-........-..............--.....................T---..........................................................

est1S_10A ....-...--........-........-..............--.....................----..........................................................

est1S_10AA ....-...--........-........-..............--.....................----..........................................................

est1S_s9A ....-...--........-........-..............--.....................T---..........................................................

est1S_s9AA ....-...--........-........-..............--.....................T---..........................................................

jai1S_s1A ....-...--........-........A..............--.....................TT--..........................................................

jai1S_s1B ....-...--........-........-..............--.....................TT--..........................................................

jai1S_s2A ....-...--........-........-..............--.....................TT--..........................................................

jai1S_s2B ....-...--........-........-.....C........--.....................T---..........................................................

jai1S_s3A ....-...--........-A....G..-..............--.....................T---..........................................................

jai1S_s3B ....-...--........-........A..............--.....................TT--..........................................................

jai1S_s4A ....-...--........-A....G..-..............--.....................T---..........................................................

jai1S_s4B ....-...--........-........A..............--.....................----..........................................................

jai1S_1A ....-...--........-........A..............--.....................----..........................................................

jai1S_1B ....-...--........-........A..............--.....................TT--..........................................................

jai1S_2A ....-...--........-........A..............--.....................TT--..........................................................

jai1S_2B ....-...--........-........-..............--.....................T---..........................................................

jai1S_3A ....-...--........-........A..............--.....................T---..........................................................

jai1S_3AA ....-...--........-........A..............--.....................T---..........................................................

jai1S_4A ....-...--........-........A..............--.....................T---......T...................................................

jai1S_4B ....-...--........-........A..............--.....................TTT-..........................................................

jai1S_5A ....-...--........-........-..............--.....................T---..........................................................

jai1S_5AA ....-...--........-........-..............--.....................T---..........................................................

jai1S_6A ....-...--........-........A..............--.....................T---..........................................................

jai1S_6AA ....-...--........-........A..............--.....................T---..........................................................

jai1S_7A ....-...--........-A....G..-..............--.....................T---..........................................................

jai1S_7B ....-...--........-........A..............--.....................T---..........................................................

jai1S_8A ....-...--........-........A..............--.....................T---..........................................................

jai1S_8AA ....-...--........-........A..............--.....................T---..........................................................

ter_1A ....-...--........-A....G..-..............--.....................T---..........................................................

ter_1B ....-...--........-........A..............--.....................T---..........................................................

ter_2A ....-...--........-........A..............--.....................T---..........................................................

ter_2B ....-...--........-........-..............--.....................T---..........................................................

ter_3A ....-...--........-........A..............--.....................T---..........................................................

ter_3B ....-...--........-A....G..-..............--.....................----..........................................................

ter_4A ....-...--........-A....G..-..............--.....................TT--..........................................................

ter_4AA ....-...--........-A....G..-..............--.....................TT--..........................................................

ter_5A ....-...--........-........A..............--.....................TTTT..........................................................

ter_5B ....-...--...G....-........A..............--.....................T---..........................................................

ter_6A ....-...--........-........-..............--.....................T---..........................................................

ter_6B ....-...--........-........-..............--.....................T---..........................................................

ter_7A ....-...--........-........A..............--.....................TT--..........................................................

ter_7B ....-...--........-........A..............--.....................T---..........................................................

ter_8A ....-...--........-........A..............--.....................T---......T...................................................

ter_8B ....-...--........-........A..............--.....................T---..........................................................

ter_9A ....-...--........-........A..............--.....................T---..........................................................

ter_9B ....-...--........-A....G..-..............--.....................TT--..........................................................

ter_10A ....-...--........-A....G..-..............--.....................T---..........................................................

ter_10B ....-...--........-........A..............--.....................----..........................................................

ter_11A ....-...--........-........A..............--.....................T----.........................................................

ter_11AA ....-...--........-........A..............--.....................T----.........................................................

ter_12A ....-...--........-........A..............--.....................TT--..........................................................

ter_12AA ....-...--........-........A..............--.....................TT--..........................................................

pan_4B ....--.G--........-........-..T...........--.....................----..........................................................

pan_4A ....--.G--........-........-..............--.....................----..........................................................

pan_8A ....--.G--........-........-..T...........--.....C..............-----..........................................................

pan_8AA ....--.G--........-........-..T...........--.....C..............-----..........................................................

pan_2B ....--.G--........-........-..T...........--.....................----..........................................................

pan_2A ....--.G--........-........-..............--.....................----..........................................................

pan_18B ....--.G--........-........-..T...........--.....................----..........................................................

pan_18A ....--.G--........-........-..T...........--.....C..............-----..........................................................

pan_14A ....--.G--........-........-..............--.....................----..........................................................

pan_14AA ....--.G--........-........-..............--.....................----..........................................................

pan_9B ....--.G--........-........-..............--.....................----..........................................................

pan_9A ....-...--........-........-..............--.....................----..........................................................

pan_7A ....--.G--........-........-..T...........--.....................----..........................................................

pan_7AA ....--.G--........-........-..T...........--.....................----..........................................................

pan_6B ....--.G--........-........-..T...........--.....C..............-----..........................................................

pan_6A ....--.G--........-........-..T...........--.....................T---.................C........................................

pan_17B ....--.G--........-........-..T...........--.....................T---..........................................................

pan_17A ....--.G--........-........-..............--.....................----..........................................................

pan_16B ....--.G--........-........-..T...........--.....................T---..........................................................

pan_16A ....--.G--........-........-..............--.....................----..........................................................

pan_15B ....--.G--........-........-..T...........--....................GT---......T...................................................

pan_15A ....--.G--........-........-..T...........--.....................T---..........................................................

pan_12B ....--.G--........-........-..T...........--.....................T---..........................................................

pan_12A ....--.G--........-........-..T...........--....................GT---......T...................................................

pan_11B ....--.G--........-........-..T...........--.....................T---..........................................................

pan_11A ....--.G--........-........-..T...........--.....................T---..........................................................

pan_21B ....-...--........-........-..............--.....................T---..........................................................

pan_21A ....--.G--........-........-..T...........--.....................----..........................................................

pan_19B ....-...--........-........-..............--.....................T---..........................................................

pan_19A ....-...--........-........-..............--.....................----..........................................................

pan_13B ....--.G--........-........-..............--.....................----............C.............................................

pan_13A ....-...--........-........-..............--.....................T---..........................................................

nat_7B ....--.G--........-........-..............--....................-----..........................................................

nat_7A ....--.G--........-........-..T...........--.....................----..........................................................

nat_6B ....--.G--........-........-..T...........--.....................----..........................................................

nat_6A ....--.G--........-........-..T...........--.....................----..........................................................

nat_5B ....--.G--........-........-..T...........--.....................----..........................................................

nat_5A ....--.G--........-........-..T...........--.....................----..........................................................

nat_4B ....--.G--........-........-..............--.....................----..........................................................

nat_4A ....--.G--........-........-..............--.....................----..........................................................

nat_3B ....--.G--........-........-..............--.....................----..........................................................

nat_3A ....--..--........-........-..............--....................-----..........................................................

nat_2B ....--.G--........-........-..............--.....................----..........................................................

nat_2A ....--.G--........-........-..T...........--.....................----..........................................................

nat_11B ....--.G--........-........-..T...........--.....................----..........................................................

nat_11A ....--..--........-........-..............--....................-----..........................................................

nat_10B ....--.G--........-........-..............--.....................----..........................................................

nat_10A ....--.G--........-........-..T...........--.....................----..........................................................

nat_9B ....--.G--........-........-..T...........--....................AT---..........................................................

nat_9A ....--.G--........-...G....-..............--.....................----..........................................................

nat_8B ....--.G--........-........-..............--.....................----..........................................................

nat_8A ....--.G--........-........-..T...........--.....................T---..........................................................

nat_1B ....--.G--........-........-..T...........--...........T.........T---..........................................................

nat_1A ..C.A-..--........-........-..............--....................-----......T...................................................

nat_12B ...--..G--........-........-..T...........--.....................----..........................................................

nat_12A ...--..G--........-........-..............--.....................TT--......T...................................................

sob2S_6A ....--.G--........-........-..T...........--.....................----..........................................................

sob2S_6AA ....--.G--........-........-..T...........--.....................----..........................................................

sob2S_11A ..C.A-..--........-........-..............--....................-----......T...................................................

sob2S_11AA ..C.A-..--........-........-..............--....................-----......T...................................................

sob2S_5A ....--.G--........-........-..............--.....................----..........................................................

sob2S_5AA ....--.G--........-........-..............--.....................----..........................................................

sob2S_4B ....--.G--........-........-..............--.....................----..........................................................

sob2S_4A ....--.G--........-........-..T...........--.....................----..........................................................

sob2S_1A ....--.G--........-........-..............--.....................----..........................................................

sob2S_1AA ....--.G--........-........-..............--.....................----..........................................................

sob2S_18B ....--.G--........-........-..............--.....................----..........................................................

sob2S_18A ....--.G--........-........-..T...........--.....................----..........................................................

sob2S_17B ....--.G--........-........-..T...........--.....................----..........................................................

sob2S_17A ....--.G--........-...G....-..............--.....................----..........................................................

sob2S_15A ....--.G--........-........-..............--.....................----..........................................................

sob2S_15AA ....--.G--........-........-..............--.....................----..........................................................

sob2S_13A ....--.G--........-........-..............--.....................----..........................................................

sob2S_13AA ....--.G--........-........-..............--.....................----..........................................................

sob2S_9B ....--.G--........-........-..............--.....................----..........................................................

sob2S_9A ...--..G--........-........-..............--.....................TT--......T...................................................

sob2S_8B ....--.G--........-........-..T...........--.....................----..........................................................

sob2S_8A ....--.G--........-........-..............--.....................----..........................................................

sob2S_7B ....--..--........-........-..............--.....................----..........................................................

sob2S_7A ....--.G--........-........-..............--.....................----..........................................................

sob2S_16B ....C-..--........-........-..............--....................-----..........................................................

sob2S_16A ....--.G--........-........-..............--.....................----..........................................................

sob2S_14B ....--.G--........-........-..............--.....................----..................................................A.......

sob2S_14A ....-...--........-........-..............--.....................T---..................................................A.......

jai2S_s9B ...--..G--........-........-..T...........--.....................----..........................................................

jai2S_s8A ...--..G--........-........-..T...........--.....................----..........................................................

jai2S_2A ...--..G--........-........-..T...........--.....................----..........................................................

jai2S_2AA ...--..G--........-........-..T...........--.....................----..........................................................

jai2S_s12A ...--..G--........-........-..T...........--.....................----..........................................................

jai2S_s12AA ...--..G--........-........-..T...........--.....................----..........................................................

jai2S_s3B ...--..G--........-........-..T...........--.....................----..........................................................

jai2S_s4B ...--..G--........-........-..T...........--.....................----..........................................................

jai2S_s5B ...--..G--........-........-..............--.....................----..........................................................

jai2S_s6A ...--..G--........-........-..T...........--...........T.........----..........................................................

jai2S_s6AA ...--..G--........-........-..T...........--...........T.........----..........................................................

jai2S_s4A ...--..G--........-........-..T...........--.....................----..........................................................

jai2S_s7A ...--..G--........-........-..T...........--.....................----..........................................................

jai2S_s10A ...--..G--........-........-..T...........--.....................TT--..........................................................

jai2S_s10B ...--..G--........-........-..T...........--.....................----..........................................................

jai2S_s11B ...--..G--........-...G....-..............--.....................----..........................................................

jai2S_s11A ...--..G--........-........-.CT...........--...........T.........----..........................................................

jai2S_1B ...--..G--........-........-.CT...........--...........T.........----..........................................................

jai2S_1A ...--...--........-........-..............--.....................----..........................................................

jai2S_s8B ...--...--........-........-..............--.....................----..........................................................

jai2S_s5A ...--..G--........-........-..T...........--.....................T----.........................................................

jai2S_s7B ...--..G--........-........-..T...........--...........T.........T---..........................................................

jai2S_s9A ...--..G--........-.......C-..T...........--.....................T---..........................................................

jai2S_s3A ...--..G--........-........-..T...........--.....................----..........................................................

est2S_26A ...--..G--........-........-..T...........--.....................----..........................................................

est2S_25A ...--..G--........-........-..T...........--.....................----..........................................................

est2S_28A ...--..G--........-........-..T...........--.....................----..........................................................

est2S_27B ...--..G--........-........-..T...........--.....................----..........................................................

est2S_23A ...--..G--........-........-..T...........--.....................----..........................................................

est2S_22A ...--..G--........-........-..T...........--.....................----..........................................................

est2S_32A ...--..G--........-........-..T...........--.....................----..........................................................

est2S_32AA ...--..G--........-........-..T...........--.....................----..........................................................

est2S_27A ...--..G--........-........-..............--.....................----..........................................................

est2S_24A ...--..G--........-........-..............--.....................----..........................................................

est2S_29A ...--..G--........-........-..............--.....................----..........................................................

est2S_23B ...--..G--........-........-..............--.....................----..........................................................

est2S_25B ...--..G--........-........-..............--.....................----..........................................................

est2S_31A ...--..G--........-........-..T...........--.....................GT--..........................................................

est2S_29B ...--..G--........-........-..T...........--.....................----..........................................................

est2S_28B ...--..G--........-........-..T...........--.....................----..........................................................

est2S_24B ...--...--........-........-..............--.....................----..........................................................

est2S_22B ...--..G--........-........-..............--.....................----..........................................................

est2S_31B ...--..G--........-........-..............--.....................TT--......T...................................................

est2S_26B ....-...--........-........-..............--.....................T---......T...................................................

est2S_s1A ....--.G--........-........-..T...........--.....................----..........................................................

est2S_s1B ....--.G--........-........-..T...........--.....................----..........................................................

est2S_s3A ..C.A-..--........-........-..............--....................-----......T...................................................

est2S_s3B ....--.G--........-........-..T...........--.....................----..........................................................

est2S_s4A ....--.G--........-........-..T...........--.....................----..........................................................

est2S_s4B ....--.G--........-........-..............--.....................----..........................................................

est2S_s5A ....--.G--........-........-..T...........--.....................----..........................................................

est2S_s5AA ....--.G--........-........-..T...........--.....................----..........................................................

est2S_s6A ..C.A-..--........-........-..............--....................-----......T...................................................

est2S_s6B ....--.G--........-........-..T...........--.....................----..........................................................

est2S_s7A ....--..--........-........-..............--.....................----..........................................................

est2S_s7AA ....--..--........-........-..............--.....................----..........................................................

mar_1A ....--.GA--.......-........-..T...........--.....................----..........................................................

mar_1AA ....--.GA--.......-........-..T...........--.....................----..........................................................

mar2A ....--.GA--.......-........-..T...........--.....................----..........................................................

mar_2B ....AG.----.......-........-..............--.....................----..........................................................

mar_11A ....AT..--........-........-..............TG.....................TT--..........................................................

mar_11B ....AG.----.......-........-..............--.....................----..........................................................

cruzi_1A ....AT.C--..G.....-........-..............TG.....................----..........................................................

cruzi_1B ....AT.C--..G.....-........-..T...........TG.....................T---..........................................................

cruzi_2A ....AT.C--..G.....-........-..............TG.....................----..........................................................

cruzi_2B ....AT.C--..G.....-........-..............TG.....................T---..........................................................

cruzi_3A ....AT.C--..G.....-........-..............TG.....................T---..........................................................

cruzi_3AA ....AT.C--..G.....-........-..............TG.....................T---..........................................................

cruzi_4A ....AT.C--..G.....-........-..............TG.....................----..........................................................

cruzi_4B ....AT.C--..G.....-........-..............TG............T........----..........................................................

cruzi_5A ....AT.C--..G.....-........-..............TG.....................TT--..........................................................

cruzi_5AA ....AT.C--..G.....-........-..............TG.....................TT--..........................................................

cruzi_6A ....AT.C--..G.....-........-..T...........TG.....................T---..........................................................

cruzi_6B ....AT.C--..G.....-........-..............TG.....................T---..........................................................

cruzi_7A ....AT.C--..G.....-........-..............TG.....................T---..........................................................

cruzi_7AA ....AT.C--..G.....-........-..............TG.....................T---..........................................................

cruzi_8A ....AT.C--..G.....-........-..............TG.....................T---..........................................................

cruzi_8AA ....AT.C--..G.....-........-..............TG.....................T---..........................................................

cruzi_9A ....AT.C--..G...T.-........-..............TG.....................TT--..........................................................

cruzi_9B ....AT.C--..G.....-........-..T...........TG.....................T---..........................................................

cruzi_10A ....AT.C--..G.....-........-..T...........TG.....................T---..........................................................

cruzi_10B ....AT.C--..G.....-........-..............TG.....................T---..........................................................

cruzi_11A ....AT.C--..G.....-........-..............TG.....................TT--..........................................................

cruzi_11B ....AT.C--..G.....-........-..............TG.....................T---..........................................................

cruzi_12A ....AT.C--..G.....-........-..............TG.....................T---..........................................................

cruzi_12B ....AT.C--..G.....-........-..T...........TG.....................TT--..........................................................

pseudo_14A ...T--..--........-........--............A--.............C.......----..........................................................

pseudo_14AA ...T--..--........-........--............A--.............C.......----..........................................................

pseudo_25A ...T--..--........-........--............A--.............C.......----..........................................................

pseudo_25AA ...T--..--........-........--............A--.............C.......----..........................................................
