# Supplementary material for: Fixed Differences in the paralytic Gene Define Two Lineages within the Lutzomyia longipalpis Complex Producing Different Types of Courtship Songs
Source: PLoS One. 2012 Sep 7;7(9):e44323. doi: 10.1371/journal.pone.0044323 (PMC3436889; doi:10.1371/journal.pone.0044323)
Supplement: Table S1 — Distribution of the 40 haplotypes found among Lu. longipalpis and Lu. cruzi samples, segregating sites within a 251 bp non-recombinant fragment and number of sequences represented in each sample. (DOC) [file pone.0044323.s002.doc]

**Table S1: Distribution of the 40 haplotypes found among *Lu. longipalpis* and *Lu. cruzi* samples, segregating sites within a 251-bp non-recombinant fragment and number of sequences represented in each sample.**

|  | **Number of sequences represented in each sample** | | | | | | | | | | | | |  |
| --- | --- | --- | --- | --- | --- | --- | --- | --- | --- | --- | --- | --- | --- | --- |
| **H** | **S1S** | **S2S** | **J1S** | **J2S** | **E1S** | **E2S** | **Lap** | **Jac** | **Nat** | **Pan** | **Ter** | **Mar** | ***Lu. cruzi*** | **Total** |
| **H1** |  |  |  |  |  |  |  | 1 |  |  |  |  |  | 1 |
| **H2** |  |  |  |  |  |  |  | 3 |  |  |  |  |  | 3 |
| **H3** |  |  |  |  |  |  |  | 2 |  |  |  |  |  | 2 |
| **H4** | 2 |  |  |  | 19 |  |  | 2 |  |  |  |  |  | 23 |
| **H5** |  |  |  |  |  |  |  | 1 |  |  |  |  |  | 1 |
| **H6** |  |  |  | 1 |  |  |  |  |  |  |  |  |  | 1 |
| **H7** |  |  |  | 1 |  |  |  |  |  |  |  |  |  | 1 |
| **H8** |  |  |  | 2 |  |  |  |  |  |  |  |  |  | 2 |
| **H9** |  |  |  |  |  |  |  |  | 1 |  |  |  |  | 1 |
| **H10** |  | 2 |  |  |  |  |  |  | 1 |  |  |  |  | 2 |
| **H11** |  | 1 |  |  |  | 1 |  |  |  | 5 |  | 1 |  | 8 |
| **H12** |  |  |  |  |  |  |  |  |  |  |  | 5 |  | 5 |
| **H13** |  | 19 |  |  |  | 8 |  |  | 17 | 26 |  |  |  | 70 |
| **H14** |  | 1 |  | 16 | 1 | 17 |  |  | 2 |  |  |  |  | 37 |
| **H15** |  |  |  |  |  |  |  |  |  |  |  |  | 1 | 1 |
| **H16** |  | 1 |  |  |  |  |  |  |  |  |  |  |  | 1 |
| **H17** |  |  |  |  |  |  |  |  |  | 1 |  |  |  | 1 |
| **H18** |  |  |  | 1 |  |  |  |  |  |  |  |  |  | 1 |
| **H19** |  |  |  |  |  | 1 |  |  |  |  |  |  |  | 1 |
| **H20** | 2 |  | 3 |  |  |  |  |  |  |  | 5 |  |  | 10 |
| **H21** |  |  |  |  |  |  |  |  |  |  | 1 |  |  | 1 |
| **H22** |  |  |  |  |  |  |  |  |  |  | 1 |  |  | 1 |
| **H23** |  |  |  |  |  |  |  |  |  |  | 1 |  |  | 1 |
| **H24** |  |  | 1 |  |  |  |  |  |  |  |  |  |  | 1 |
| **H25** | 1 |  |  |  |  |  |  |  |  |  | 1 |  |  | 2 |
| **H26** |  |  |  |  |  |  | 1 |  |  |  |  |  |  | 1 |
| **H27** | 4 |  | 6 |  |  |  | 4 | 10 |  |  | 2 |  |  | 26 |
| **H28** | 17 |  | 11 |  |  |  | 9 |  |  |  | 12 |  |  | 49 |
| **H29** |  |  |  |  |  |  |  | 1 |  |  |  |  |  | 1 |
| **H30** | 1 |  |  |  |  |  |  |  |  |  |  |  |  | 1 |
| **H31** | 1 |  |  |  |  |  |  |  |  |  |  |  |  | 1 |
| **H32** | 1 |  | 2 |  |  |  | 14 |  |  |  |  |  |  | 17 |
| **H33** |  |  |  |  |  |  |  | 1 |  |  |  |  |  | 1 |
| **H34** | 2 |  |  |  |  |  |  |  |  |  |  |  |  | 2 |
| **H35** |  |  | 1 |  |  |  |  |  |  |  |  |  |  | 1 |
| **H36** |  |  |  |  |  |  |  |  |  |  | 1 |  |  | 1 |
| **H37** |  |  |  |  |  |  |  |  |  |  |  |  | 1 | 1 |
| **H38** |  |  |  |  |  |  |  |  |  |  |  |  | 20 | 20 |
| **H39** |  |  |  |  |  |  |  |  |  |  |  |  | 1 | 1 |
| **H40** |  |  |  |  |  |  |  |  |  |  |  |  | 1 | 1 |

**H: Haplotype, S1S: Sobral 1S, S2S: Sobral 2S, J1S: Jaíba 1S, J2S: Jaíba 2S, E1S: Estrela 1S, E2S: Estrela 2S, Lap: Lapinha, Jac: Jacobina, Nat: Natal, Pan: Pancas, Ter: Teresina, Mar: Marajó.**
